# Annotations

**Published:** 1891-01-31

**Authors:** 


### Annotations.

**Physical Education.**

The *British Medical Journal* published last week the first of a series of articles on "The Place of Physical Education." It is time that medicine shook off some of her old and narrow traditions, and recognised her duty to the healthy, as well as her interest in the sick. "Not play only, nor work only," says the journal, "but that combination of play and work, which alone can make men out of what would otherwise develope into shiftless idlers, or precocious pedants, is recognised as the ideal of true education." And again : "It seems that almost instinctively, the school boy has evolved, as the complement of intellectual labour, some sort of system, whose value lies in its disciplined exercise of the body ; and that, long before we realised its nature, mankind had empirically laid its hand upon the truth that physical training is, equally with mental training, an integral factor of all true education. . . . The first essential is to rid the mind of all notions of an essential antagonism between work and play, to substitute the revelation that these two forms of exercise are mutually important factors in education, and thereupon to graft the corollary that each demands not merely its due recognition, but an equal share of forethought, method, and application." We do not quote these things because they are new, but because they are true, and because they are spoken *ex cathedra* in an authoritative quarter. Truths that are obvious to common sense are sometime very common-place, and secure little regard and less obedience. But if they are taken up by those who have an acknowledged right to speak, they become at once the exponents and compellers of duty. Medical men, of all persons, are the priests and the prophets of the body; and medical newspapers gather up and shape into sentences of concentrated wisdom the ripe experience that is common to the medical world. So much of that experience as belongs strictly to medicine should be confined to medicine ; but there is some of it which belongs more to the world than to medicine. Such is the knowledge which appertains to the education of the body ; and we are glad that the *British Medical Journal* is recognising one of its wider duties, and is undertaking to teach the parents of the world a kind of wisdom which they greatly need to learn.

**About Breakfast.**

"This morning," says Pepys, "Mr. Shepley and I did eat our breakfast at Mrs. Harper's (my brother John being with me) upon a cold turkey pie and a goose." "This morning" was January 6th, and, doubtless, a cold, winter day ; and yet Master Pepys and his brother John, with Mr. Shepley, sat down, evidently with satisfactory appetites, to a breakfast of cold goose and cold turkey pie. It is to be feared that many a London man of the present age sitting down to such a breakfast would fare poorly. A Norfolk squire might, perhaps, manage to satisfy his hunger at a table of that kind, though even he would require to hunt at least four days a-week, and to wear a blue ribbon. Breakfast is rather a failure as a meal with town men. That ought not to be ; there is something wrong when a man is not vigorously hungry in the morning. Where is the fault? Is it in the late dinner? Not in the dinner, probably, so much as in what is drunk at dinner : in that, and in the nervous strain of the times. Of course Pepys dined in the middle of the day, and equally of course he had supper. It does not matter whether we dine in the middle of the day or in the evening, so long as we dine judiciously. But the man who has much work to do, and particularly brain work, cannot dine in the middle of the day. If he does, he must make up his mind to lose at least an hour of his most valuable time. A light lunch at midday, with no stimulant stronger than a cup of coffee or a bottle of ginger ale, is the suitable thing. But this must on no account be used as a substitute for dinner. He who lunches in this way at midday must dine in the evening, and dine well The business man should dine at half-past six, or at latest at half-past seven. The lazy man may dine when he likes. The man who has earned his dinner should have a good one—not heavy, but nutritious ; not too elaborate, but well selected and well cooked. He should drink, if possible, only one kind of wine, and that a light wine, sparkling or still. Spirits and beer he should avoid. If he cannot afford wine, he should try some of the non-alcoholic beverages, which are very well manufactured now. Dinner should be the last meal of the day, except for those who cannot sleep without a little food in their stomachs. These may take a cup of cocoa, with a little thin bread and butter, just at the moment of going to bed. If attention be paid to these suggestions very few people will fail to be hungry at breakfast. They may not rise to Pepys' cold goose and turkey pie, but they will find no difficulty in settling accounts with a good slice of ham and a couple of fresh eggs. Nobody should feel happy who persistently fails to eat a good breakfast.

**The Hospitals Commission Again.**

The Lords' Commission on Hospitals has recommenced its sittings. It may be well to give the names of the members. They are : The Archbishop of Canterbury, Earl Cadogan, the Earl of Winchelsea, the Earl of Lauderdale, Earl Spencer, Earl Cathcart, the Earl of Kimberley, Lord Zouche of Haryngworth, Lord Saye and Sele, Lord Clifford of Chudleigh, Lord Sandhurst, the Earl of Erne, Lord Lamington, the Earl of Arran, Lord Monkswell, and Lord Thring. Lord Sandhurst is, as before, President of the Commission. There is no doubt whatever that these noble Lords mean to do well, both by the hospitals and by the public. There are two ways of getting at any facts you may desire to know. The one way is that used by the judicious parent or the sympathetic counsellor of youth ; the other is the method of the law court and of the cross-examining counsel. All public bodies of investigators are inclined to adopt the law method ; they think it is the surest way of ferreting out the facts. But we submit that everything depends upon the kind of persons that are to be examined. With a wily criminal, trained and experienced in all the shifts and dodges of the courts, perhaps the only way of arriving even at a partially clear presentment of facts is by the free use of the methods of the Old Bailey. But when all the witnesses are not only persons of undoubted character and honourable motives, but are as anxious as the noble Lords themselves to show to the Commission and the public the case exactly as it stands with hospitals, it is clear that Old Bailey methods are not for a moment to be thought of. The Lords' Committee must remember that their work is as far removed as possible from that of the trial of criminals. On the contrary, their one business is to investigate the methods of various honourable institutions ; to compare one method with another ; to suggest that the best method which they can discover, if there be a best, shall be adopted universally ; and if there be not a best, to try to construct one out of the most approved elements in each existing method. The Lords have to learn, as other people have learnt, that there is often found "much cry" where there is very "little wool." If a king commit a fault it is blazoned abroad throughout the whole earth; if a beggar commit the same fault nobody either knows or cares to know anything at all about it. Similarly, if a newspaper writer, eager for a sensational paragraph for his journal, can hear of any really or seemingly unkind or dishonourable action done at such a noble and philanthropic institution as a hospital, he rushes into print with it in the maddest haste ; whereas, if the same thing were done by a small business company, or by a workmen's club, he would not put pen to paper about the matter. The Lords must remember these things. They have no legal peers to guide them as they would have if they were sitting in their own chamber. They must, therefore, be all the more cautious, and all the more judicial. Above all, they must use the common sense of experienced men of the world. Their own homes will furnish them with miniatures of what hospitals ought more or less to be. Let them use the same principles and methods as they would if they were investigating the domestic affairs of a hundred of their peers. Let them remember that a gentleman is always a gentleman, and will always give as fair and just a view of facts as his mental powers will furnish him with. Let them remember, too, that women are always women, and that they cannot help being both more impulsive and less judicial than men. Let them remember also that the lower we go in the scale of education, the more do passion and self-interest usurp the place of reason, and the less can witnesses be relied upon to give clear, succinct, and exact presentations of facts as they are. Now, what is our apology for offering these suggestions to the Lords' Committee? It is this : That we have been behind the scenes for many years—they have not.

